# Combined Activity of the Redox-Modulating Compound Setanaxib (GKT137831) with Cytotoxic Agents in the Killing of Acute Myeloid Leukemia Cells

**DOI:** 10.3390/antiox11030513

**Published:** 2022-03-08

**Authors:** Muhammed Burak Demircan, Peter C. Mgbecheta, Anne Kresinsky, Tina M. Schnoeder, Katrin Schröder, Florian H. Heidel, Frank D. Böhmer

**Affiliations:** 1 Institute of Molecular Cell Biology, CMB, Jena University Hospital, 07745 Jena, Germany; muhammedburak.demircan@pei.de (M.B.D.); peter.mgbecheta@gmail.com (P.C.M.); anne.kresinsky@gmx.de (A.K.); 2 Innere Medizin II, Hämatologie und Onkologie, Jena University Hospital, 07747 Jena, Germany; tina.schnoeder@uni-greifswald.de (T.M.S.); florian.heidel@uni-greifswald.de (F.H.H.); 3 Leibniz Institute on Aging—Fritz Lipman Institute, 07745 Jena, Germany; 4 Molecular Biotechnology and Gene Therapy, Paul-Ehrlich-Institut, 63225 Langen, Germany; 5 Innere Medizin C, Universitätsmedizin Greifswald, 17475 Greifswald, Germany; 6 Institute for Cardiovascular Physiology, Goethe University, 60590 Frankfurt am Main, Germany; schroeder@vrc.uni-frankfurt.de

**Keywords:** acute myeloid leukemia (AML), reactive oxygen species (ROS), NADPH oxidase 4 (NOX4), CRISPR/Cas9-mediated deletion, inhibitor, Setanaxib, GKT137831

## Abstract

Acute myeloid leukemia (AML) cells harbor elevated levels of reactive oxygen species (ROS), which promote cell proliferation and cause oxidative stress. Therefore, the inhibition of ROS formation or elevation beyond a toxic level have been considered as therapeutic strategies. ROS elevation has recently been linked to enhanced NADPH oxidase 4 (NOX4) activity. Therefore, the compound Setanaxib (GKT137831), a clinically advanced ROS-modulating substance, which has initially been identified as a NOX1/4 inhibitor, was tested for its inhibitory activity on AML cells. Setanaxib showed antiproliferative activity as single compound, and strongly enhanced the cytotoxic action of anthracyclines such as daunorubicin in vitro. Setanaxib attenuated disease in a mouse model of FLT3-ITD driven myeloproliferation in vivo. Setanaxib did not significantly inhibit FLT3-ITD signaling, including FLT3 autophosphorylation, activation of STAT5, AKT, or extracellular signal regulated kinase 1 and 2 (ERK1/2). Surprisingly, the effects of Setanaxib on cell proliferation appeared to be independent of the presence of NOX4 and were not associated with ROS quenching. Instead, Setanaxib caused elevation of ROS levels in the AML cells and importantly, enhanced anthracycline-induced ROS formation, which may contribute to the combined effects. Further assessment of Setanaxib as potential enhancer of cytotoxic AML therapy appears warranted.

## 1. Introduction

Reactive oxygen species (ROS) comprise a number of radical or non-radical compounds, which are either produced by cell metabolism or generated by cell-external mechanisms such as exposure to ionizing radiation. Important ROS molecules are the superoxide anion (O_2_^•−^) and hydrogen peroxide (H_2_O_2_). Among cellular sources of ROS are mitochondria, in which O_2_^•−^ is produced as a side product of the respiratory chain activity, and NADPH oxidases (NOXs), which produce either O_2_^•−^ or H_2_O_2_ (NOX4). Cellular ROS production is important for physiological signaling processes, e.g., signaling of growth factor receptor-tyrosine kinases involving reversible oxidation of counteracting protein-tyrosine phosphatases. Supraphysiological ROS levels causing oxidative stress associated with damage to cellular macromolecules and tissues, have been associated with several pathologies, including *Diabetes mellitus*, neurodegenerative diseases, ischemia-reperfusion injury, e.g., related to ischemic stroke and chronic inflammatory conditions such as chronic obstructive pulmonary disease (COPD) [1,2,3]. High levels of reactive oxygen species (ROS) have also been observed in cancer cells of tumors of different origin and have, in several cases, been causally linked with malignancy. For example, ROS-associated damage to mitochondrial DNA has been linked to breast cancer progression [4]. To enable cell survival in the presence of high ROS levels, elevated activity of ROS detoxifying mechanisms is likewise common in cancer cells. Two different pharmacological strategies have been attempted to exploit these features for a selective cancer therapy: Inhibition of ROS formation, using antioxidants or—more specifically—inhibitors of ROS-generating enzymes, or promotion of ROS production to cytotoxic levels. Until now, none of these approaches has advanced to clinical use. However, cytotoxic activity of several established cancer drugs has been partially linked to the promotion of excessive ROS formation [5,6,7]. 

Acute myeloid leukemia (AML) is a heterogenous cancer entity based on rather different genetic traits [8,9]. It has often unfavorable prognosis, related to its occurrence at higher age, therapy-limiting comorbidities and adverse cytogenetics. The commonly used ‘induction chemotherapy’ involves treatment with anthracyclines (daunorubicin, idarubicin or mitoxantrone) and the antimetabolite cytarabine, followed by either consolidation chemotherapy or, if indicated, allogeneic stem-cell transplantation [10,11]. A subset of 25–30% of patients are diagnosed with a mutation in the gene encoding Fms-like tyrosine kinase 3 (FLT3), rendering FLT3 constitutively active. The predominant mutation-type is described as ‘internal tandem duplications (ITD)’ of amino acid sequence in a region of the kinase which, in the physiological setting, regulates activity negatively. Presence of the ITD mutation causes strong FLT3 activation and mislocalization into intracellular compartments such as the ER. FLT3-ITD mutations are associated with an aggressive disease and particularly poor prognosis [12,13,14,15,16]. This prompted the development of FLT3 tyrosine kinase inhibitors, of which several have been recently approved for clinical treatment of AML with FLT3 mutations [17,18]. The first approved inhibitor midostaurin represented one of the first targeted therapies for AML [19]. However, as 5-year overall survival rates for AML continue to be below 40%, especially in the elderly, there is a clear need for new therapeutic options.

Over the last few years, several groups including our own, have demonstrated elevated ROS levels in AML cells, leading to the speculation that altered ROS metabolism may permit novel therapeutic approaches [20,21,22,23,24]. In FLT3-ITD-positive cells, part of the ROS elevation is driven by FLT3 activity, involving a heightened level and activity of the ROS-producing enzyme NADPH-oxidase 4 (NOX4) [25]. We therefore considered the possibility that inhibition of NOX4 activity may be a strategy of targeting FLT3-ITD-positive AML cells. 

Setanaxib (GKT137831) has initially been described as a dual NOX1/4 inhibitor with potency in the submicromolar range [26]. Because NOX4 has been established as a mediator of different chronic inflammatory and fibrotic pathologies, the orally available compound has been assessed in vivo for the treatment of related diseases including fibrotic kidney disease, idiopathic pulmonary fibrosis, and primary biliary cholangitis [27]. Even rather high doses of Setanaxib are well tolerated in animals and humans, and clinical trials with Setanaxib for treatment of primary biliary cholangitis have advanced to stage II/III (ClinicalTrials.gov identifier: NCT05014672). Relatively little work has previously been carried out to assess the potential activity of Setanaxib against cancer. Previous experiments from our own lab revealed a moderate antiproliferative activity against FLT3-ITD-positive AML cells [25]. 

In the present study, we have further investigated the effects of Setanaxib on AML cells. In particular, we focused on a potential additive value of Setanaxib when combined with cytotoxic agents. We noted a strong synergy of anthracyclines with Setanaxib for decreasing viability of FLT3-ITD-positive AML cells in vitro, and also therapeutic activity of Setanaxib in a mouse model of FLT3-ITD driven myeloproliferation. Surprisingly, neither the antiproliferative activity of Setanaxib nor the cytotoxic activity when combined with daunorubicin was related to NOX4 inhibition. CRISPR/Cas9 engineered *NOX4* knockout cells appeared equally sensitive to the compound. Inhibition by Setanaxib was also not confined to FLT3-ITD-positive cells and may be based on enhanced rather than on attenuated ROS production. Given its advanced state of clinical development and excellent safety profile, exploring Setanaxib in combination with cytotoxic agents for the treatment of AML appears of interest. 

## 2. Material and Methods

### 2.1. Cell Lines and Reagents

MV4-11, MOLM13, HL60, and OCI-AML3 cells were obtained from the German Collection of Microorganisms and Cell Lines (DSMZ) and cultivated in RPMI 1640 medium supplemented with 10% heat-inactivated fetal bovine serum (FBS), 100 µL Plasmocin^TM^ per 500 mL, and passaged every 2–3 days. FLT3-ITD-expressing 32D cells and Ba/F3 cells were obtained as described earlier [28,29,30] and cultivated in RPMI 1640 medium supplemented with 10% FBS, 2.5 ng/mL murine IL-3, and 1 mM sodium pyruvate. HEK293 cells with tetracycline (tet)-inducible NOX4 expression [31] were kindly provided by Prof. K.H. Krause (University of Geneva, Geneva, Switzerland), and cultivated in DMEM medium supplemented with 10% FBS. Cell lines were routinely checked for absence of mycoplasma by PCR assay.

To obtain FLT3-ITD/MA9 or MA9 cells, 2 × 10^6^ bone-marrow (BM) cells of C57/BL6 *Flt3*^ITD/ITD^ knockin [32] or C57/BL6 wildtype mice were transduced by standard techniques (details available on request) with pMSCV-MLL-AF9/GFP (retroviral vector expressing the human MLL-AF9 fusion gene and an IRES-GFP, kindly provided by Prof. S. Armstrong, Dana-Farber Cancer Institute, Boston, MA, USA). In case of FLT3-ITD cells, 5 × 10^4^ GFP^+^ cells were transplanted two days later via lateral tail vein injection into sublethally irradiated (7 Gy) C57BL/6 ‘incubator mice’. Moribund mice were sacrificed and BM cells were isolated and cultured in RPMI medium without any cytokines. MA9 cells were directly obtained from pMSCV-MLL-AF9/GFP-transduced BM cells by cultivation in medium supplemented with IL3. Primary human AML cells were obtained with informed consent of patients from peripheral blood as described earlier [25] (permission ethic board of Jena University Hospital 4653-04/16). 

Setanaxib (GKT137831) was obtained from Selleckchem (Munich, Germany), or MedChemExpress (Monmouth Junction, NJ, USA). Daunorubicin, doxorubicin, and cytarabine were obtained from the Pharmacy, Jena University Hospital (Jena, Germany). AC220 was a kind gift from Prof. Siavosh Mahboobi (University of Regensburg, Regensburg, Germany). Midostaurin was from Sigma Aldrich (Taufkirchen, Germany). Concentrated compound stock solutions were generated in DMSO and diluted with medium as required, the final DMSO concentration in the cell culture was 0.05%. 

### 2.2. Generation of Genetically Modified Cell Lines

Details of cell line modification will be reported elsewhere (Demircan et al., manuscript submitted). In brief, MOLM13, MV4-11, Ba/F3 cells, and HEK293 cells (with tet-inducible NOX4) with stable Cas9 expression were generated by transduction with lentiviral particles expressing Cas9 (Streptococcus pyogenes gene in lentiCas9-Blast plasmid, #52962, Addgene Cambridge, MA, USA) and cell selection with blasticidin using standard techniques, and subsequent clonal selection was employed for high Cas9 levels, monitored by immunoblotting. For designing efficient sgRNA vectors, the software GPP sgRNA Designer (Broad Institute, Cambridge, MA, USA) was used, and corresponding oligonucleotides were ligated into the ipUSEPR vector (vector and sgLuci control construct kindly provided by Prof. S. Armstrong, Dana-Farber Cancer Institute, Boston, MA, USA). Cells were transduced with lentiviral particles expressing the targeted sgRNA or luciferase-targeting sgRNA (control) and selected with puromycin. Knockout of the target gene(s) was validated using a genomic PCR approach for human/mouse *NOX4/Nox4*, or immunoblotting for p22-phox. Efficiency of NOX4-directed sgRNAs was also assessed using the stable NOX4-overexpressing HEK293 cells described under Section 2.1 and immunoblotting. Details of the used sequences and detection tools will be presented in a separate paper (Demircan et al., manuscript submitted) and are also available upon request.

### 2.3. Proliferation and Apoptosis Assays

Cell proliferation was determined by cell counting with a Neubauer hemocytometer as described in the figure legends. Alternatively, viable cell amounts were measured using Cell Titer-Blue (Promega, Walldorf, Germany). To this end, 5 × 10^3^ or 3 × 10^4^ (for murine cell lines or human AML cell lines, respectively) cells were seeded in 100 µL growth medium per well into 96-well black plates (Greiner, Frickenhausen, Germany), and complemented with 25 µL growth medium with different concentrations of drugs. After incubation for 3 days, 25 µL Cell Titer-Blue reagent was added into each well and plates were incubated for another 3 h. The fluorescence signal was measured with a TECAN Infinite 200 plate reader at excitation and emission wavelengths of 540 nm and 610 nm, respectively.

Apoptosis assays were performed using the Annexin-V method. Cells were treated with the drug or drug combinations for 48 h and detection of apoptotic cells was carried out with a PE annexin-V kit (AB_2869265, BD Biosciences, Heidelberg, Germany) according to the instructions of the manufacturer. 

### 2.4. Animal Experiments

Animal experiments were performed with the permission of authorities of the country of Thuringia (license # UKJ-18-012) in agreement with European law for animal welfare. FLT3-ITD and green-fluorescent protein (GFP)-expressing 32D cells (2 × 10^6^ or 5 × 10^5^, see figure legends) in phosphate-buffered saline (PBS) were injected into the tail vein of C3H/HeJ mice (Jackson Laboratory, Bar Harbor, ME, USA). Setanaxib was applied by oral gavage for 9 consecutive days as described previously [25]. Doxorubicin solution for patient use (Doxo Cell 150, STADAPHARM GmbH, Bad Vilbel, Germany, 2 mg/mL) was diluted with sterile 150 mM NaCl to 0.6 mg/mL and 5 µL/g was injected intraperitoneally at day 3, 4, and 5 after transplantation of cells. Mice were monitored for signs of morbidity and were sacrificed at day 9 or 10 post transplantation. The whole bone marrow (BM) containing invaded GFP+ 32D-FLT3-ITD cells was harvested from the tibia, femur, and hip. To this end, the bones were removed from the mice, the muscles were stripped off using a sterile paper towel, and the cleaned bones were placed in a Petri dish. The bones were cut with scissors and the marrow was obtained by flushing the bone interiors with PBS using a syringe. The suspension was collected and passed through a 70 μm cell strainer. Spleens were harvested and smashed by passing the tissue through a 70 μm cell strainer with a syringe pestle. The suspensions were centrifuged and blood cell lysis was performed using BD Pharm Lyse™ lysing solution (BD Biosciences, Heidelberg, Germany) according to the instructions of the manufacturer. The percentage of GFP-positive cells in BM or spleen was subsequently determined by flow cytometry using a BD FACSCanto^TM^ flow cytometer. The gating strategy is illustrated with example dot-plots for spleen cell analysis in Supplementary Figure S1 (Online Supplement). Data were analyzed using FlowJo software (TreeStar Inc., Ashland, OR, USA).

### 2.5. Immunoblotting and Reactive Oxygen Assays

SDS-PAGE and immunoblotting were performed as described earlier [25]. The following antibodies were used: anti-pY694 STAT5 (ab32364, Abcam, Cambridge, UK); anti-pY589/591 FLT3 (#3464), anti-pS473 AKT (#4060), anti-pERK1/2 137F5 (#4695), anti-AKT (#9272), and anti-ERK1/2 3A7 (#9107) from Cell Signaling (Frankfurt, Germany); anti-FLT3 (sc-480) and anti-STAT5a/b rabbit polyclonal (sc-835) from Santa Cruz (Heidelberg, Germany).

Reactive oxygen species were determined using the dye DCFDA/H_2_DCFDA (ab113851, Abcam, Cambridge, UK) or the Cellular ROS Assay Kit (Deep Red) (ab186029, Abcam, Cambridge, UK) and subsequent FACS analysis according to the instructions of the manufacturer.

### 2.6. Determination of Synergism and Statistics

Synergism of the drug effects in viability and apoptosis assays was determined with the method of Chou [33] using the program Calcusyn (Biosoft Corporation, Cambridge, UK). Statistic tests were performed with GraphPadPrism (GraphPad Software Inc., La Jolla, CA, USA) and specified in the figure legends.

## 3. Results

### 3.1. Setanaxib Has Inhibitory Activity on Growth of AML Cells In Vitro

**Figure 1:**
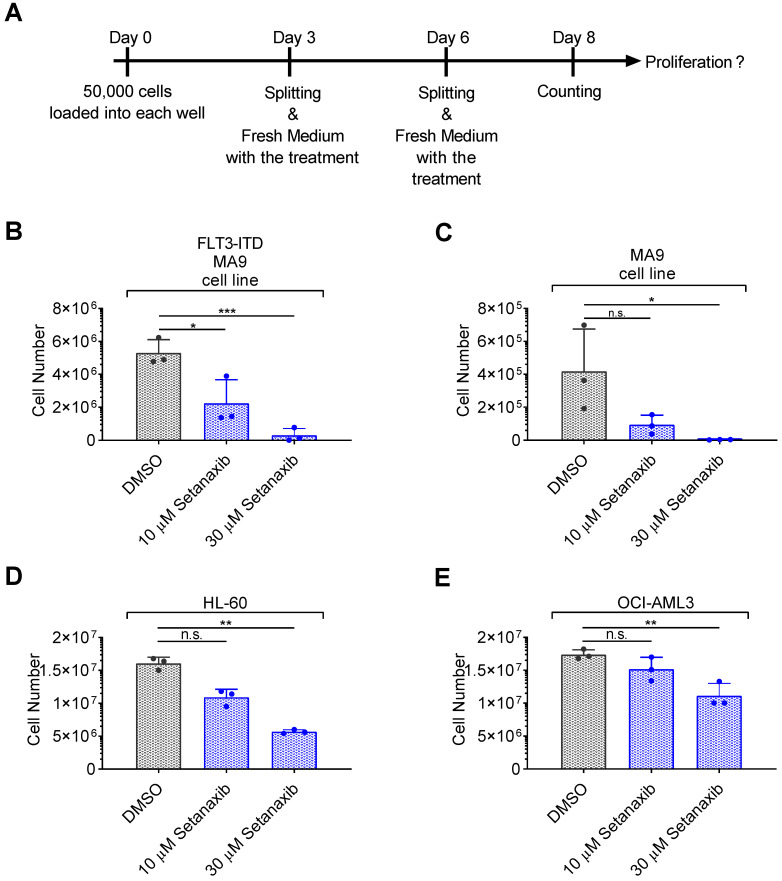
Setanaxib inhibits the proliferation of AML cell lines. The effect of Setanaxib on the proliferation of different leukemic cells was assessed. Experimental conditions for treatment with Setanaxib were as indicated in (**A**), and results are shown in (**B**–**E**). FLT3-ITD/MLL-AF9-transformed cells (**B**) or MLL-AF9-transformed cells (**C**) were obtained by the transduction of murine bone-marrow stem cells of FLT3-ITD knock-in mice or of wildtype C57BL/6 mice, respectively, with MLL-AF9. Only MLL-AF9-transformed cells were cultivated in presence of IL3. HL-60 and OCI-AML3 are human AML cell lines which do not harbor mutated FLT3. Cells were seeded at low density (50,000 cells/well) in 24-well plates and treated with two different doses of Setanaxib as indicated in (**A**). At day 8, cells were counted using a hemocytometer. The individual experiments were normalized to DMSO control. Three independent experiments (with technical triplicates) were conducted; error bars represent mean ± SD. (n.s.—not significant, * *p* < 0.05, ** *p* < 0.01, *** *p* < 0.001 comparisons with DMSO controls by two-tailed *t*-test).

Previously published data had shown moderate inhibitory activity of Setanaxib on the proliferation of FLT3-ITD-positive AML cell lines, including MV4-11 cells, MOLM13 cells, and stable FLT3-ITD-transduced murine 32D cells [25]. Using a more elaborate proliferation assay (Figure 1A), we observed complete inhibition for both FLT3-ITD/MLL-AF9 or only MLL-AF9-positive leukemia cell lines generated by oncogene transduction of murine bone marrow stems cells (Figure 1B,C). Human AML HL60 or OCI-AML3 cells, which do not harbor mutated FLT3 (Figure 1D,E), were also potently inhibited. To avoid potential artefacts through interference of Setanaxib with mitochondrial ROS metabolism, cell counting was used rather than metabolic assays. Consistent with our earlier observations [25], the required Setanaxib doses for inhibition were in the micromolar range.

### 3.2. Setanaxib Is Synergistic with Daunorubicin in FLT3-ITD-Positive Cells

**Figure 2:**
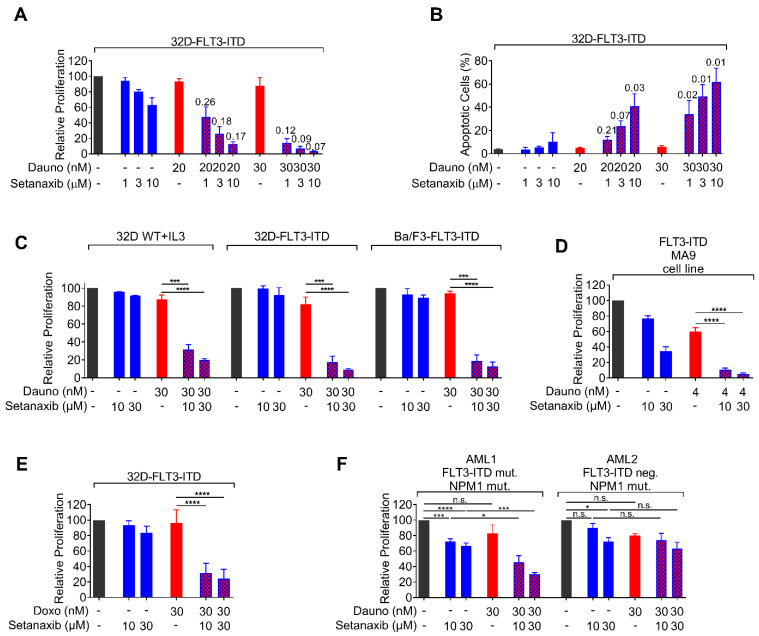
Effect of the single and combined drug treatments on proliferation/viability and induction of apoptosis in AML cells. (**A**) 32D-FLT3-ITD cells were seeded in 96-well plates, single compounds or combinations of compounds (daunorubicin, Setanaxib, or combinations) were added, and the proliferation/viability was assessed by Cell Titer Blue assay after 72 h. The fluorescent signal, which was directly proportional to the number of viable cells, was measured in a plate reader at 540/610 nm (excitation/emission, respectively, RFU-relative fluorescence units). (**B**) Apoptosis rate was measured by FACS analysis using Annexin V/7-AAD staining after 48 h of single or combined treatments with daunorubicin and Setanaxib. The combination index was determined using the software Calcusyn. The obtained ‘combination index (CI)’ numbers are shown above the bars. Three independent experiments were conducted; error bars represent mean ± SD. (**C**) Comparison of synergistic effects in wildtype (WT) FLT3-expressing 32D-cells, FLT3-ITD-expressing 32D- cells, and Ba/F3-FLT3-ITD cells. Note that the cultivation of WT-FLT3 32D cells requires addition of IL-3 to the medium. (**D**) Synergy of Setanaxib and daunorubicin in a murine leukemic cell line transformed with FLT3-ITD and MLL-AF9 obtained as indicated in the legend to Figure 1. (**E**) Synergy of Setaxanib with doxorubicin in 32D-FLT3-ITD cells. The individual experiments were normalized to DMSO controls. Three independent experiments (with technical triplicates) were conducted; error bars represent mean ± SD. (**** *p* < 0.0001 by two-tailed *t*-test). Note that for clarity in (**C**–**E**) only selected statistical comparisons are shown to emphasize the synergistic effects of Setanaxib addition to a dose of anthracycline, which has limited effect alone. (**F**) Synergy of Setanaxib with daunorubicin in primary human AML cells. Primary patient PBMCs were isolated by Ficoll density gradient separation. Cells (100,000 cells/well) were treated with drugs or their combinations as indicated. Proliferation was assessed as in (**A**). The individual experiments were normalized to DMSO controls. Error bars represent mean ± SD. Statistical analyses were carried out using one-way ANOVA with Tukey’s post-test. (n.s.—not significant, * *p* < 0.05 and *** *p* < 0.001).

We subsequently tested the synergy of Setanaxib in combinations with the cytotoxic and targeted drugs commonly used to treat AML, daunorubicin, cytarabin, and midostaurin. For simplicity, we used here the metabolic assay CellTiter Blue, which gave identical results with cell counting in side-by-side comparison using Setanaxib as a single drug (data not shown). To adjust suitable doses, effective dose ranges of the cytotoxic drugs were assessed with human MV4-11 cells and murine FLT3-ITD-expressing 32D cells and found to be in the previously reported range of active concentrations (Supplementary Figure S2, Online Supplement). We observed additive inhibitory effects in particular for the combination of daunorubicin with Setanaxib as shown for FLT3-ITD-positive murine cells in Figure 2A,C–E, but also in murine 32D cells harboring wildtype FLT3. Side-by-side comparison revealed similar sensitivity of wildtype FLT3-expressing 32D cells and FLT3-ITD-expressing 32D cells, with a slightly stronger response to the combination of both drugs in the latter (Figure 2C). A limitation of these experiments is, however, that wildtype FLT3-expressing cells require the presence of IL-3 for survival, and this cytokine activates similar pathways as FLT3-ITD. While Setanaxib alone had minor effects on the induction of apoptosis, high levels of apoptosis were induced by the drug combination (Figure 2B). These effects were synergistic according to data analysis using the method of Chou [33]. Notably, we observed synergistic inhibition of Setanaxib with daunorubicin also in a FLT3-ITD/NPM1-mutated primary human AML cell sample, while a FLT3-wildtype/NPM1-mutated cell sample appeared less sensitive (Figure 2F). While this finding shows susceptibility of primary human cells to the drug combination, the assessment of larger numbers of AML cell samples will be required to establish a potential difference in the sensitivity of primary human AML cells with different FLT3 status. An overview of results regarding the observed synergism of Setanaxib containing combinations with different cell lines is presented in Table 1. Taken together, Setanaxib enhances the efficiency of classical AML therapeutics in vitro.

### 3.3. Setanaxib Has Efficiency In Vivo

**Figure 3:**
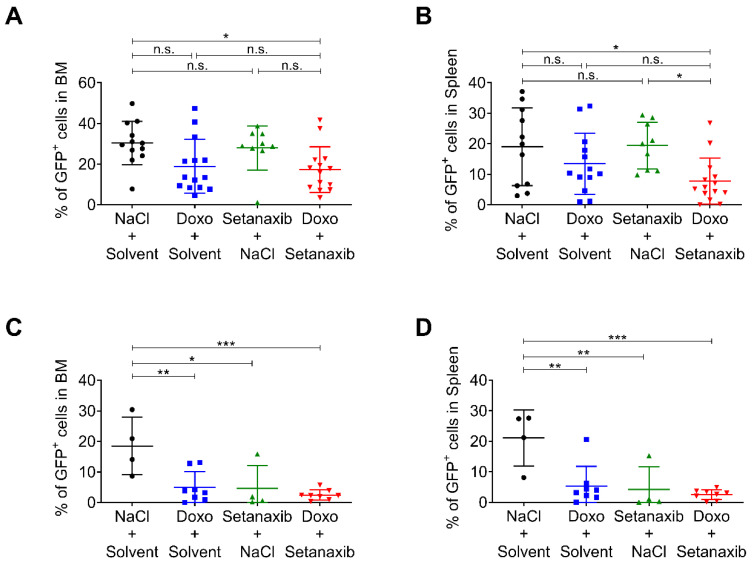
Effect of single and combined drug treatments with Setanaxib and doxorubicin in vivo. (**A**,**B**) 2 × 10^6^ GFP-expressing 32D FLT3-ITD cells were injected in C3H/HeJ mice via the tail vein, and respective drug treatments were applied. Setanaxib was given at 40 mg/kg body weight by daily gavage for 9 days, and doxorubicin at 3 mg/kg body weight by i.p. injection at day 3, 4, and 5 post-transplantation. Mice in independent experiments (two cohorts) were sacrificed 9- or 10-days post-transplantation. The percentage of GFP-positive cells was determined by flow cytometry in (**A**) BM and (**B**) spleen. (**B**,**C**) 5 × 10^5^ GFP-expressing 32D FLT3-ITD cells were injected in C3H/HeJ mice and respective drug treatments were applied as in (**A**,**B**). Mice in independent experiments (two cohorts) were sacrificed 10 days post-transplantation. The percentage of GFP-positive cells was determined by flow cytometry in (**C**) BM and (**D**) spleen (*n* = 4–8 per group, mean ± SD). Statistical analyses were carried out using one-way ANOVA with Tukey´s post-test. (n.s.—not significant, * *p* < 0.05, ** *p* < 0.01, and *** *p* < 0.001).

We used a mouse model of FLT3-ITD-induced myeloproliferation to test the efficiency of the drug combination in vivo. In this model, FLT3-ITD-positive 32D cells are injected into syngeneic C3H/HeJ mice, which develop an early onset and fatal myeloproliferation. This model operates with a relatively high burden of inoculated tumor cells (2 × 10^6^ per mouse), leading to death within less than two weeks. When used as monotherapy, Setanaxib had beneficial effects with moderately prolonged survival and reduction in tumor burden at death [25]. For reasons of animal welfare, we applied this model in a modified form. Instead of survival, the tumor burden was measured as the abundance of GFP-positive cells in bone marrow and spleen. To assess for potential additive value, we used doxorubicin, an anthracycline that can be applied intraperitoneally in mice [34]. Doxorubicin was titrated to identify a dose with limited efficiency (3 mg/kg body weight by i.p. injection at day 3, 4, and 5 post-transplantation). Setanaxib at 40 mg/kg daily showed minor efficiency when used as oral monotherapy in mice injected with 2 × 10^6^ tumor cells (Figure 3A,B). Doxorubicin as single compound appeared to reduce tumor burden, but the effect was not significant. Only the combination of doxorubicin and Setanaxib attenuated the disease to a significant extent, as measured by a reduction in GFP-positive cells in bone marrow (BM) and spleen (Figure 3A,B) compared with only solvent-treated animals. When using fewer numbers of inoculated cells (5 × 10^5^), Setanaxib had an inhibitory effect as a single drug (Figure 3C,D). In the only Setanaxib group, 3/4 mice showed less than 2% of GFP-positive cells in BM and spleen while mice in the control group showed around 20% of GFP-positive cells in both compartments. Doxorubicin on its own was likewise potent in reducing the tumor burden as a single drug. Under these conditions, no significant difference was detectable when comparing the effects of the drug combination with that of doxorubicin treatment alone. We only observed a trend of improved efficiency using the combination of both drugs when compared with the solvent control. Taken together, the experiments indicate the potential of Setanaxib to attenuate the myeoloproliferative disease in this model alone and in combination with anthracycline therapy.

### 3.4. Effects of Setanaxib Are Independent of FLT3-Signaling, Presence of NOX4, or ROS Quenching

**Figure 4:**
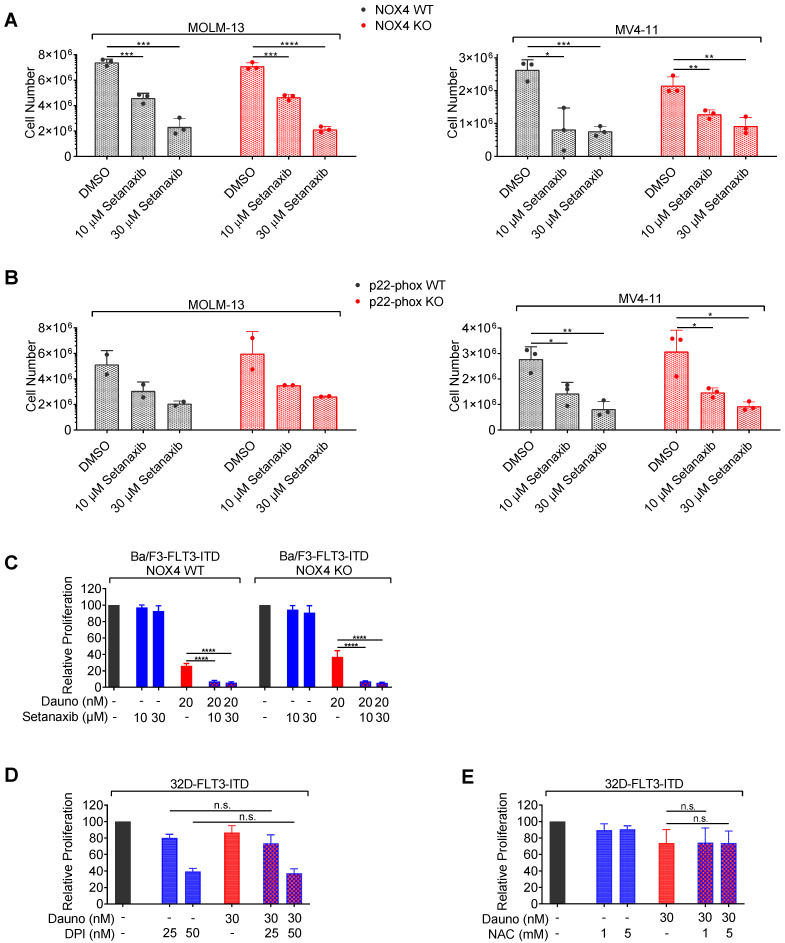
Assessment of a potential mechanism for Setanaxib effects. (**A**,**B**) Effect of Setanaxib on the proliferation of human AML cells with *NOX4* or *p22-phox* knockout. KO human AML cells were obtained using CRISPR/Cas9 technology. The cell lines stably express Cas9 and were either transduced with control sgRNA (sgLuci, designated WT) or sgRNA targeting *NOX4* or *p22-phox*, respectively (designated KO). Absence of the targeted gene was detected as described in Materials and Methods. The experimental setup for the drug treatments and assay of proliferation were as in Figure 1A,B. Cells were counted using a hemocytometer at day 8. Mean ± SD of cell treatments of two to three independently sgRNA-transduced cell batches is presented. (**C**) Comparison of synergy of Setanaxib with daunorubicin in Ba/F3-FLT3-ITD cells harboring Cas9 and transduced with sgLuci (WT) or sg*Nox4* (KO). Treatments were performed as in Figure 2 and proliferation/viability was assessed by Cell Titer Blue assay. The individual experiments were normalized to DMSO controls. Mean ± SD (with technical triplicates) for cells from three independent transductions with sgRNA is shown. (**D**,**E**) The 32D-FLT3-ITD cells were subjected to single or combined drug treatments with daunorubicin, diphenyleneiodonium (DPI) or N-acetylcysteine (NAC) as indicated for 72 h, and proliferation/viability was assessed by Cell Titer Blue assay. Three independent experiments (in triplicate) were conducted; error bars represent mean ± SD. Statistical analyses were carried out using two-tailed *t*-test (n.s.—not significant, * *p* < 0.05, ** *p* < 0.01, *** *p* < 0.001, and **** *p* < 0.0001).

Next, we sought to assess the mechanism of Setanaxib-mediated decrease in cell viability. Setanaxib has initially been described as a NOX1/4 inhibitor. However, recent data provided first evidence that Setanaxib may not directly inhibit any of the NOX family members [35,36]. To investigate the involvement of NOX4 inhibition in the observed antiproliferative effects on AML cells, we tested Setanaxib on the human AML cell lines MV4-11 and MOLM13, either expressing NOX4 or after genetic inactivation of NOX4 by CRISPR/Cas9 technology. No difference in antiproliferative activity of the drug was observed regardless of whether the cells contained NOX4 or not, rendering inhibition by affecting enzymatic activity of NOX4 unlikely (Figure 4A). We further analyzed cells in which the common subunit of NOX1, 2, 3, and 4, p22-phox was either present or deleted using CRISPR/Cas9. Elimination of p22-phox is expected to abolish the activity of all four NOX enzymes [37]. Again, Setanaxib was equally inhibitory for cells lacking p22-phox, confirming that its effect is not mediated through the inhibition of enzymatic activity of either NOX1 or NOX4 (Figure 4B). Its inhibitory capacity, independent from the presence of NOX enzyme activity, was specifically also observed in combination with daunorubicin, when using FLT3-ITD-transduced Ba/F3 cells (Figure 4C). Taken together, neither antiproliferative activity of Setanaxib nor synergy with daunorubicin in AML cell lines appears to be causally related to the inhibition of NOX1/4 enzymatic activity.

Inhibitory activity of Setanaxib and its observed synergy with anthracyclines may, however, be based on general antioxidant activity. We addressed this issue indirectly by testing two compounds with an established general activity on redox metabolism: Diphenyleneiodonium (DPI), a flavoprotein inhibitor and general NOX inhibitor, and N-acetylcysteine (NAC), a general antioxidant. When we used these compounds in concentrations known to exert antioxidant effects, DPI as single compound inhibited cell growth (Figure 4D), while NAC only had a little effect (Figure 4E). Importantly, no synergy with daunorubicin could be observed (Figure 4D,E). 

**Figure 5:**
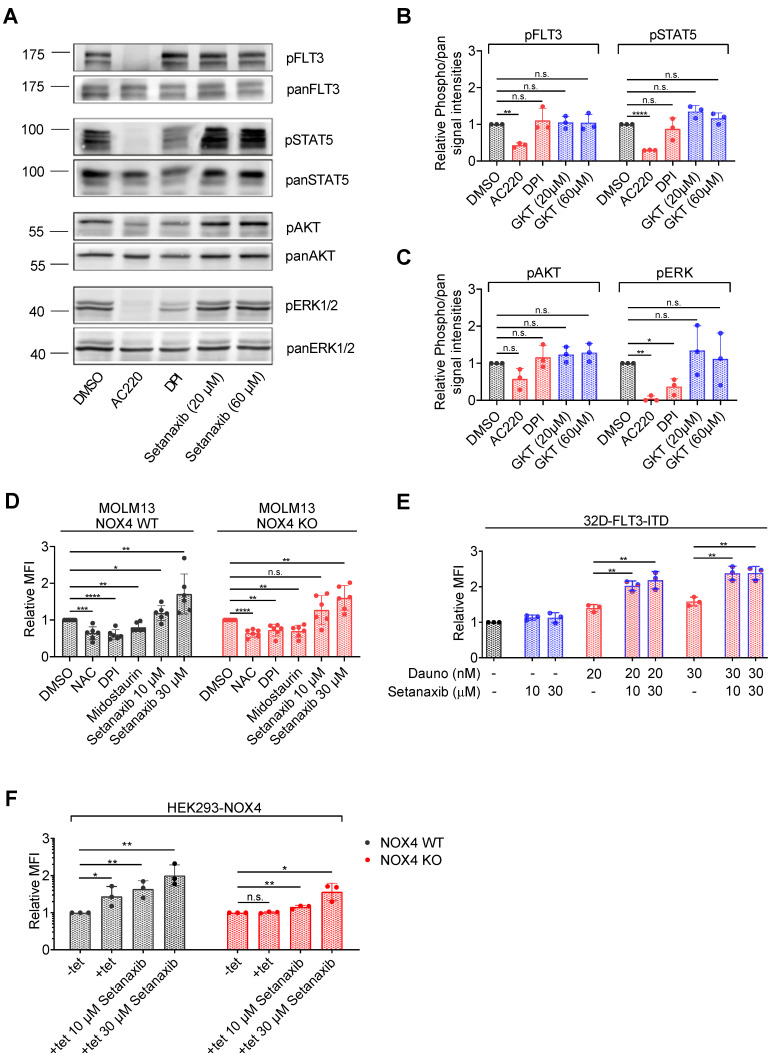
Setanaxib promotes ROS formation elicited by daunorubicin. (**A**–**C**) MV4-11 cells were treated with the indicated concentrations of the FLT3-ITD inhibitor AC220 (quizartinib, positive control), the general NOX inhibitor diphenyleneiodonium (DPI), or Setanaxib for 4 h in serum-free medium. Thereafter, cells were lysed and lysates subjected to immunoblotting for assessment of pathway activation. Blots were probed with activation-specific antibodies to pFLT3 (pY589/591), pSTAT5 (pY694), pAkt (Ser473), or pErk1/2 (Thr202/Tyr204) antibodies. Subsequently, blots were stripped and comparable loading was validated by reprobing of the membranes with antibodies against FLT3, STAT5, Akt, or Erk as indicated. (**A**) Representative result. (**B**,**C**) Quantification of three independent experiments for the indicated signaling molecules. Setanaxib (GKT137831) is abbreviated as GKT. Error bars represent mean ± SD. Statistical analyses were carried out with two-tailed *t*-test. (**D**) MOLM-13 cells were treated with 5 mM NAC (positive control), 500 nM of the general NOX inhibitor diphenyleneiodonium (DPI), 40 nM of the protein kinase inhibitor midostaurin, or 10 or 30 µM Setanaxib for 24 h. Thereafter, cells were stained with H_2_DCFDA for 30 min and ROS levels were quantified on the flow cytometer (MFI, mean fluorescence intensity). Error bars represent mean ± SD, (*n* = 3). Statistical analyses were carried out using two-tailed *t*-test. (**E**) The 32D-FLT3-ITD cells were treated with the indicated concentrations of the drugs for 24 h. Thereafter, cells were stained with ROS Deep Red dye for 30 min and ROS levels were quantified by flow cytometry. The experiment was conducted three times independently. Error bars represent mean ± SD, (*n* = 3). Statistical analyses were carried out using two-tailed *t*-test. (**F**) HEK293 cells with tetracycline (tet)-inducible NOX4 overexpression and engineered to constitutively express Cas9 were kept uninduced (−tet) or were induced (+tet) to overexpress NOX4 and were mock-treated with solvent or were treated with Setanaxib as indicated. ROS formation was scored with H_2_DCFDA as in (**D**). Error bars represent mean ± SD, (*n* = 3). Statistical analyses were carried out using two-tailed *t*-test (n.s.—not significant, * *p* < 0.05, ** *p* < 0.01, *** *p* < 0.001, and **** *p* < 0.0001).

Although the activity of Setanaxib did not appear to be restricted to FLT3-ITD-positive AML cells, we also assessed the potential effects of Setanaxib on FLT3-ITD-mediated oncogenic signaling. MV4-11 cells were treated with Setanaxib and lysates were analyzed by immunoblotting for activation of FLT3, STAT5, AKT, and ERK based on the presence of their phosphorylated forms. AC220 (quizartinib), a potent FLT3 kinase inhibitor [38], and DPI were used as controls. As shown in Figure 5A–C, AC220 reduced activation of the assessed signaling molecules strongly. DPI showed a significant inhibitory effect on ERK activation (Figure 5C). Of note, Setanaxib did not significantly inhibit any of these signaling events even at the high concentration of 60 µM (Figure 5A–C).

Anthracyclines such as daunorubicin induce apoptosis in cancer cells by causing DNA damage. However, they also contribute to the elevation of ROS. This ROS induction can contribute to the induction of cancer cell apoptosis [39,40]. Therefore, we considered that Setanaxib, despite its proposed targets NOX1 and 4, may rather modulate ROS metabolism and affect daunorubicin-induced ROS elevation. Setanaxib treatment resulted in dose-dependent elevation of ROS in MOLM13 cells as indicated by H_2_DCFDA fluorescence (Figure 5D). Again, this effect was independent of NOX4 expression. NAC, DPI, and midostaurin caused a reduction in ROS levels, as described before [22,41]. Importantly, daunorubicin enhanced ROS levels, and the combination of Setanaxib and daunorubicin showed additive effects in enhancing ROS (Figure 5E). Similar observations were made in HEK293 cells, engineered to inducibly overexpress NOX4. NOX4 overexpression caused elevated ROS formation, which was further enhanced by Setanaxib. CRISPR/Cas9-mediated knockout of NOX4 in these cells abolished tet-induced ROS elevation; however, a dose-dependent induction of ROS formation by Setanaxib was likewise visible (Figure 5F). These findings support that Setanaxib can promote ROS formation, and that this effect is independent from NOX4. While the exact mechanisms of Setanaxib-induced ROS formation remain so far elusive, synergy in elevated and critical ROS production may play a role for the observed synergy in cytotoxicity.

## 4. Discussion and Conclusions

In this study, we have found that Setanaxib, an inhibitor with proposed activity against NOX1 and 4 function, shows antiproliferative effects on AML cells. This effect was prominently seen in leukemia cells harboring the oncogenic driver FLT3-ITD, but also in cell lines which do not carry an FLT3 mutation. Importantly, in both the FLT3-ITD-expressing and wildtype FLT3 cells it strongly synergizes with cytotoxic agents, in particular the anthracyclines such as daunorubicin. Surprisingly, these effects are independent of NOX1/4 expression and may instead be mediated by enhancement of anthracyclin-induced ROS production possibly through an unknown pathway of modulating ROS metabolism.

Recent studies have questioned the target specificity of Setanaxib for NOX1/4 and suggested that the compound may affect ROS metabolism through other mechanisms such as inhibition of peroxidase [35,36]. We have used genetic deletion using CRISPR/Cas9 to assess the relevance of NOX4 as a potential target for the antiproliferative activity of Setanaxib in AML cells. Setanaxib was used in many studies and the observed effects considered being via NOX4 or NOX1 inhibition. However, thorough validation using genetic deletion of *NOX* genes in combination with inhibitor treatment is lacking in these studies. 

In a recent study showing cytotoxic activity of Setanaxib on liver cancer cells in vitro, a potentially interesting mechanism of action has been proposed: Treatment with Setanaxib fostered mitochondrial ROS formation and caused cell death, which was at least in part mediated by high ROS levels [42]. As we have likewise found elevated ROS formation in AML cells upon Setanaxib treatment, a mitochondrial origin of elevated ROS may be considered. Testing this hypothesis as well as assessing the potential causal link of ROS formation to cytotoxicity require further investigations.

While moderate antiproliferative effects of Setanaxib on AML cells were reported earlier [25], the strong synergy with cytotoxic agents was unexpected and novel. Antiproliferative effects of Setanaxib could also be observed in vivo. It attenuated disease as a single compound in mice injected with 5 × 10^5^ tumor cells. In mice injected with 2 × 10^6^ tumor cells, only the combination of Setanaxib with doxorubicin attenuated disease compared with solvent-treated mice. Overall, the observed effects using the syngeneic FLT3-ITD 32D/C3H/HeJ mouse model, in which disease progression is very rapid, were however quite limited. Exploring the in vivo effects of the drug and its combination with anthracyclines further, e.g., in experiments using patient-derived xenograft models is clearly warranted.

Further assessment of Setanaxib for possible treatment of AML appears interesting and reasonable. Notably, this compound is in the advanced stages of clinical development. Early clinical trials have suggested a low toxicity profile of the compound [43]. Moreover, Setanaxib has good oral bioavailability. To date, five phase I and three phase II clinical studies have been carried out with Setanaxib and no safety signal and no dose limiting toxicity have been observed. Doses up to 800 mg/day were earlier found to be safe compared to placebo in a 24-week phase II trial in PBC patients (NCT03226067). Even dosing as high as 1600 mg/d has recently been tested without identifying any dose-limiting toxicity (phase I, ClinicalTrials.gov identifier: NCT04327089) [44]. In a phase II trial in patients with diabetic nephropathy, the patients in the Setanaxib-treated arm finished the 12-week treatment period with even fewer adverse events compared to the placebo arm but did not reach the primary clinical endpoint [26] (NCT03740217, NCT02010242). In a phase II trial on primary biliary cholangitis, Setanaxib was successful in reaching its primary and secondary efficacy endpoints (NCT03226067). Other phase II clinical trials using Setanaxib are ongoing for kidney disease in type 1 diabetes, and for idiopathic pulmonary fibrosis [45]. A trial (phase II/III) for primary biliary cholangitis is currently planned (ClinicalTrials.gov identifier: NCT05014672). Given the toxicity of induction chemotherapy especially in elderly AML patients, the addition or sequential use of the well-tolerated and orally available compound Setanaxib may allow the reduction in cytotoxic drugs or its use with low-dose chemotherapy or hypomethylating agents. A further interesting aspect, which needs further exploration, is the possibility that synergy of anthracycline drugs with Setanaxib may extend to other tumor entities such as soft tissue sarcoma. 

Taken together, our data show that growth inhibition and synergistic toxicity of Setanaxib for AML cells are independent from the presence of NOX4 or the common functional NOX1-4 subunit p22-phox. Oncogenic signaling downstream of FLT3-ITD is not affected even by high concentrations of Setanaxib. Moreover, cells without activated FLT3 appear similarly sensitive to Setanaxib inhibition as FLT3-ITD-positive AML cells, supporting that the mechanism of action is not through interference with common FLT3-ITD-dependent signaling pathways. These results indicate complexity of molecular mechanisms underlying the Setanaxib-related therapeutic efficacy on different leukemia cells. Elevation of ROS levels, in particular strong enhancement of anthracycline-induced ROS elevation by Setanaxib, could be shown. While the origin of elevated ROS levels is not defined yet, high ROS levels promoted by Setanaxib in the presence of anthracyclines may result in increased toxicity and contribute to the induction of apoptosis in AML cells.

We would also like to summarize here several limitations of this study: While the cell-line data indicate that cells harboring FLT3-ITD have similar, possibly somewhat higher sensitivity to Setanaxib and to its combination with daunorubicin than cells expressing wildtype FLT3, analysis of more patient cell samples is needed for determining the effect of FLT3-ITD for susceptibility of primary AML cells. In vivo data using a syngeneic mouse model of myeloproliferative disease indicate a trend of combined activity of Setanaxib and anthracyclines, but given large assay variations and high leukemia cell burden in this model, no significant advantage of compound combination over single drugs could yet be validated. Further animal experiments are required to establish compound synergy in vivo. Finally, while our experiments exclude that the inhibitory effects of Setanaxib on cell proliferation are mediated through inhibiting NOX4, or (based on knockout of p22-phox) NOX1-3 enzymatic activity, we cannot exclude action through a non-enzymatic mechanism on NOX1, although we consider this not likely. We propose that enhanced ROS formation by treatment with Setanaxib and daunorubicin contribute to cytostatic/cytotoxic activity, but the causal role of elevated ROS formation remains yet to be proven.

## Figures and Tables

**Table 1:** Analysis of the potential synergy of the combined treatments of different AML cell lines with Setanaxib and with different cytotoxic drugs. 32D FLT3-ITD, MV4-11, or MOLM13 cells were subjected to drug treatments. Experimental conditions for cell treatments were as in Figure 2. Setanaxib was applied at 1, 3, 10 or 30 µM, cytarabine at 100 or 500 nM, daunorubicin at 20 or 30 nM, and midostaurin at 10 or 20 nM. The proliferation/viability of the cells was assessed by Cell Titer Blue assay after 72 h of treatments. Apoptosis rates were measured by FACS analysis after 48 h of treatments. The CI values were calculated with the Chou method using the software Calcusyn. A CI of <1 indicates synergism (<0.1 very strong synergism (+++++), 0.1 to 0.3 strong synergism (++++), 0.3 to 0.7 synergism (+++), 0.7 to 0.85, moderate synergism (++), 0.85 to 0.90, low synergism (+), and 0.90 to 1.1, nearly additive (±). Three independent experiments (in triplicate) were conducted.

Proliferation/Viability (Cell Titer Blue)				
	Chemotherapy drugs	32D FLT3-ITD	MV4-11	MOLM13
Setanaxib	Cytarabine	+++	±	±
	Daunorubicin	+++++	+++	+++
	Midostaurin	++++	+++	++
Apoptosis (Annexin V + 7-AAD)				
	Chemotherapy drugs	32D FLT3-ITD	MV4-11	
Setanaxib	Cytarabine	+++	±	
	Daunorubicin	+++++	+	
	Midostaurin	++++	++	

## Data Availability

The data presented in this study are available in the article and supplementary materials.

## Supplementary Materials

The following supporting information can be downloaded at: https://www.mdpi.com/article/10.3390/antiox11030513/s1, Figure S1: Analysis of GFP-positive FLT3-ITD expressing 32D cells in C3H/HeJ mice; Figure S2: Effect of standard cytotoxic drugs on proliferation/viability of MV4-11 and 32D-FLT3-ITD cells.
