# Supplementary material for: Combined Activity of the Redox-Modulating Compound Setanaxib (GKT137831) with Cytotoxic Agents in the Killing of Acute Myeloid Leukemia Cells
Source: Antioxidants (Basel). 2022 Mar 8;11(3):513. doi: 10.3390/antiox11030513 (PMC8944474; doi:10.3390/antiox11030513)
Supplement: Supplementary file 1 [file antioxidants-11-00513-s001.zip › antioxidants-1562760-supplementary.pdf]

Online Supplement

Combined activity of the redox-modulating compound Setanaxib (GKT137831) with cytotoxic agents in the killing of acute myeloid leukemia cells

Muhammed Burak Demircan [1,2,3,4], Peter C. Mgbecheta[1], Anne Kresinsky [1,3], Tina M. Schnoeder [2,5], Katrin Schröder [6], Florian H. Heidel [2, 3, 5] and Frank-D. Böhmer[1*]

[1] Institute of Molecular Cell Biology, CMB, Jena University Hospital, 07745 Jena, Germany; [2] Innere Medizin II, Hämatologie und Onkologie, Jena University Hospital, 07747 Jena, Germany; [3] Leibniz Institute on Aging—Fritz Lipman Institute, 07745 Jena, Germany; [4] Molecular Biotechnology and Gene Therapy, Paul-Ehrlich-Institut, 63225 Langen, Germany; [5] Innere Medizin C, Universitätsmedizin Greifswald, 17475 Greifswald, Germany; [6] Institute for Cardiovascular Physiology, Goethe University, 60590 Frankfurt am Main, Germany
*Corresponding author: Frank-D. Böhmer, Institute of Molecular Cell Biology, CMB, Jena University Hospital, Hans-Knöll-Strasse 2, 07745 Jena, Germany; Email i5frbo@uni-jena.de

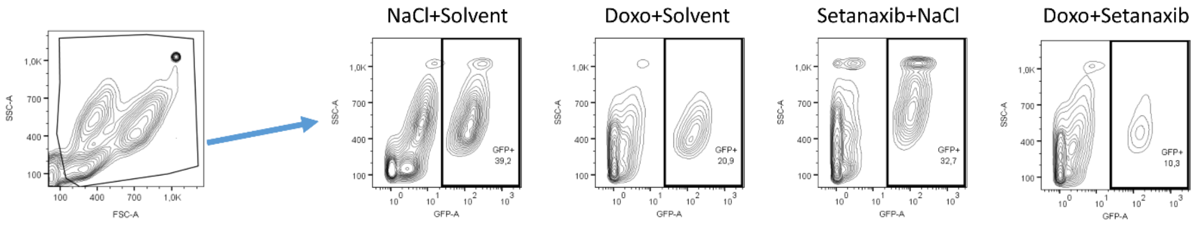

Figure S1. Analysis of GFP-positive FLT3-ITD expressing 32D cells in C3H/HeJ mice. Examples of dot-plots for FACS analysis of spleen cells corresponding to data shown in Figure 3B. Spleen cells were prepared as described in Materials and Methods (*2.4*). Left panel: Gating of cells by forward- and sideward-scatter profiles to exclude debris. Right panels: Detection of 32D-FLT3-ITD cells in the GFP channel. The control GFP+ (NaCl+Solvent) plot corresponds to the shown scatter plot. The other GFP+ plots are example measurements from animals treated with the compounds as indicated. Numbers for GFP+ in the right lower corner represent the percentage of GFP-positive cells.

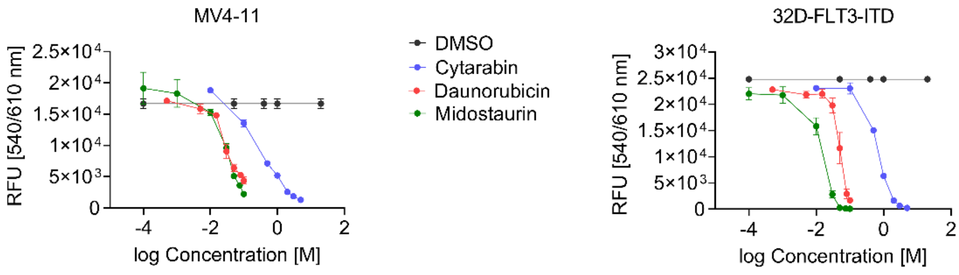

Figure S2. Effect of standard cytotoxic drugs on proliferation/viability of MV4-11 and 32D-FLT3-ITD cells. Cells were seeded in 96-well plates, compounds were added, and the proliferation/viability was assessed by Cell Titer Blue assay after 72 hours. The fluorescent signal, which was directly proportional to the number of viable cells, was measured in a plate reader at 540/610 nm (excitation/emission, respectively, RFU- relative fluorescence units).
